# Competing Distractors Facilitate Visual Search in Heterogeneous Displays

**DOI:** 10.1371/journal.pone.0160914

**Published:** 2016-08-10

**Authors:** Garry Kong, David Alais, Erik Van der Burg

**Affiliations:** 1 School of Psychology, The University of Sydney, Australia; 2 Dept. of Experimental and Applied Psychology, Vrije Universiteit Amsterdam, Netherlands; Centre de neuroscience cognitive, FRANCE

## Abstract

In the present study, we examine how observers search among complex displays. Participants were asked to search for a big red horizontal line among 119 distractor lines of various sizes, orientations and colours, leading to 36 different feature combinations. To understand how people search in such a heterogeneous display, we evolved the search display by using a genetic algorithm (Experiment 1). The best displays (i.e., displays corresponding to the fastest reaction times) were selected and combined to create new, evolved displays. Search times declined over generations. Results show that items sharing the same colour and orientation as the target disappeared over generations, implying they interfered with search, but items sharing the same colour and were 12.5° different in orientation only interfered if they were also the same size. Furthermore, and inconsistent with most dominant visual search theories, we found that non-red horizontal distractors increased over generations, indicating that these distractors facilitated visual search while participants were searching for a big red horizontally oriented target. In Experiments 2 and 3, we replicated these results using conventional, factorial experiments. Interestingly, in Experiment 4, we found that this facilitation effect was only present when the displays were very heterogeneous. While current models of visual search are able to successfully describe search in homogeneous displays, our results challenge the ability of these models to describe visual search in heterogeneous environments.

## Introduction

Visual search is a widely used task to study visual attention. This approach has revealed much about how we search for a target among distractors. For example, we know that we can find a target more efficiently when it differs significantly from the distractors in one basic attribute such as colour, orientation or size [1, 2]. These types of findings can be readily demonstrated using simple search displays, often consisting of a target and two types of distractors. As the visual search processes that apply to such simple stimuli become more familiar, however, there has been a movement towards the study of more complex stimuli, involving more distractors, often differing by more than one stimulus attribute. These can be referred to as heterogeneous displays.

Heterogeneity in visual search refers to displays where the distractors not only differ from the target, but also differ from each other; the more the distractors differ amongst themselves, the more heterogeneous we say the display is. Heterogeneity has been shown to increase the difficulty of visual search [3], but it is unclear why this occurs. It appears though, that the reason for this increase cannot be a simple generalisation of the processes already known to apply to relatively homogeneous displays often used to study visual search. Additional processes such as crowding [4] or clutter [5], have been identified as processes that play a larger role as the displays become more complex. However, the identification of such processes has been limited by the predominant experimental design in visual search research, the factorial design.

When using a factorial experimental design, one can systematically manipulate the variables of interest to draw conclusions about whether these factors affect the depending variable (i.e., search performance). Factorial designs have served the visual search community well for simple stimuli, but as we move towards heterogeneous stimuli, two limitations of factorial designs have been revealed. The first is that heterogeneous displays, by definition, consist of a multitude of factors that can influence the dependent variable, and as such, the testing of each possible combination becomes a time-consuming, if not impossible, process. As a result, designs are often *simplified*, averaging over potentially useful manipulations. For example, Nordfang and Wolfe [6] investigated visual search using a heterogeneous display consisting of distractors with 3 different features: 3 colours, 3 shapes and 3 orientations (a total of 27 possible feature combinations). To draw conclusions about participants’ search behaviour, Nordfang and Wolfe grouped the distractors based on how many features a given distractor differed from the target features. Through this grouping, they concluded that as the number of common features between the target and distractors increased, search times increased exponentially. While these overall conclusions are interesting, conclusions about the role of each possible feature combination (i.e., a specific colour, orientation and shape combination) on search could not be drawn, as it is simply infeasible to measure the effects of each feature combination in a single experiment.

The Nordfang and Wolfe [6] study can also be used to demonstrate the second limitation of using factorial designs, which is that the selection of variables to be averaged together needs to be *identified a priori*. Since this averaging is in place even in the experimental design, conclusions can only be drawn on the factors that were decided to be of interest.

For example, Nordfang and Wolfe hypothesised that the number of common features would affect visual search performance, and as such were only able to make conclusions about that. In other words, a hypothesis-driven experiment cannot be used to reliably find counter-intuitive or unexpected results. In a field as unexplored as heterogeneous displays, this is a major limitation.

In order to sidestep these problems of *identification* and *simplification* of factors of interest, we must turn to exploratory techniques to study heterogeneous displays, one of which is the genetic algorithm. Van der Burg, Cass, Theeuwes & Alais [7] recently used the genetic algorithm as an atheoretical, unbiased method to reveal people’s visual search behaviour by evolving a complex visual search display towards faster-searched displays. They used a display made up of 72 distractor lines of three orientations (0°, 10° or 90°) and 3 colours (red, green or blue). The target was always a red horizontal (i.e., 0°) line, and participants responded to the presence or absence of a gap in this line segment. 12 distractor sets were randomly created by selecting a random orientation and colour for each distractor, and each of these distractor sets was shuffled to create six search displays. The 12 distractor sets were then ranked by computing the mean reaction time of the six displays that they created. Following a ‘survival of the fittest’ principle, the four distractor sets with the fastest reaction times were selected to create a new generation of distractor sets. 12 new distractor sets were created by a process known as ‘crossover’, switching parts of the four selected distractor sets between each other; and ‘mutation’, randomly assigning another colour and/or orientation to a small number of distractors. Since the four distractor sets whose parts are used to create the new sets were selected because they created displays that were easy to search, the new distractor sets should also create displays that are easy to search. By repeating this process of testing, selection and crossover/mutation 15 times, Van der Burg et al., could trace the evolution of the distractor sets. Importantly, this evolution allowed the assessment of the contribution of each element independently, so that, for example, no averaging across orientations was required.

Consistent with the visual search literature [3, 4, 8–11], the number of red 10° lines in their displays decreased over generations, as they were the distractors most similar to the target. The results also showed a concomitant increase in the number of horizontal lines over successive generations, indicating that they facilitated faster visual search times for the red horizontal target. This latter finding is not only counterintuitive, it is also the opposite of what has been observed in previous studies exploring the role of irrelevant distractors [12–17]. Following models of visual search [3, 18, 19], the blue and green horizontally oriented lines should have hindered visual search because they share a basic feature with the target, yet they facilitated search. Importantly, by using the genetic algorithm, Van der Burg et al., were able to test each feature combination without grouping; neither was there a need to guess *a priori* which combinations would be the interesting ones.

While the genetic algorithm was shown to work in evolving displays for efficient search in Van der Burg et al.’s [7] study, there are several aspects of their implementation of the genetic algorithm that warrant further exploration. One is that all displays in their study had relatively few distractor types and displays were constrained to have the same number of lines. Consequently, when one feature combination decreased in number over generations, another one must increase to take its place. Thus, with few alternatives available, it is possible that the increase in blue and green horizontal lines was an artefact due to the decrease in red elements. In this study, we expand the number of possible feature combinations from just 8 in the Van der Burg et al., [7] study to 35. More feature combinations means an increased likelihood of an even spread of changes in distractor numbers across the various distractor types rather than a spurious clustering of changes in just a couple of types. In this way, we will disentangle the ambiguous facilitation and competition effects present in Van der Burg et al.’s [7] study. Furthermore, the expanded number of feature combinations will allow us to thoroughly study, on a finer scale, the effects of heterogeneous distractors on visual search.

## Experiment 1

In the present study, we investigate visual search in a heterogeneous display. Participants search for a big red horizontal target line among 119 distractor lines of various orientations (vertical, horizontal or ±12.5° from vertical and horizontal), colours (red, green and blue) and sizes (big and small). Participants responded as quickly and accurately as possible to the presence or absence of a gap in this target line. The gap was present in 50% of all lines, so participants could not simply search for the line with a gap. A genetic algorithm was used to examine which of the 35 possible feature combinations were either hindering or facilitating visual search

For the first generation (i.e., the first block), we created 12 distractor sets by randomly assigning an orientation, colour and size to each of the 119 distractors in the set. Each distractor set was repeated 5 times. However, the location of both target and distractors were shuffled between presentations. Subsequently, participants performed the visual search task for the 60 displays in the generation (12 distractor sets x 5 repetitions). For each of the 12 distractor sets, we calculated the median reaction time (RT) for the 5 repetitions. Following a ‘survival of the fittest’ principle, we selected the best distractor sets (i.e., those with the fastest median RTs) for reproduction. A crossover and mutation procedure was used to recombine the selected distractor sets in order to create a new generation of 12 evolved sets (see Materials and Method). Then, participants performed the search task again on these 12 evolved distractor sets (each producing 5 displays) and the cycle was repeated until participants completed 8 generations.

Given that we applied a survival of the fittest principle, we expected the distractor sets to become fitter with each generation. For this to occur, distractors that are detrimental to the search task should disappear over generations, and distractors that facilitate the search task should increase. Specifically, if the increase of non-red horizontals in the Van der Burg et al., study was due to their facilitating visual search, then these should increase over generation. Conversely, if the increase in non-red horizontal distractors in the Van der Burg et al., study was due to other distractors decreasing, then we expect to find an increase in the non-red horizontal distractors that is in line with the increase in other non-detrimental distractors, as there are now more feature combinations to spread this decrease over.

### Materials and Method

#### Ethics Statement

Written consent was obtained from each participant prior to the experiments. The experiments were approved by the local ethics committee of the University of Sydney in accordance with the Declaration of Helsinki.

#### Participants

22 participants (15 female, mean age = 20.56, ranging from 18 to 33 years) took part in this experiment. Participants had normal or corrected-to-normal vision. All participants were naïve as to the purpose of the experiment.

#### Materials

The stimuli were generated in Matlab using the Psychophysics Toolbox extensions [20–22]. They were displayed on a 19” DiamondDigital CRT monitor (100Hz refresh rate, 1024 by 768 pixels). Participants sat approximately 57 cm away from the monitor and used a standard keyboard to respond.

**Fig 1:**
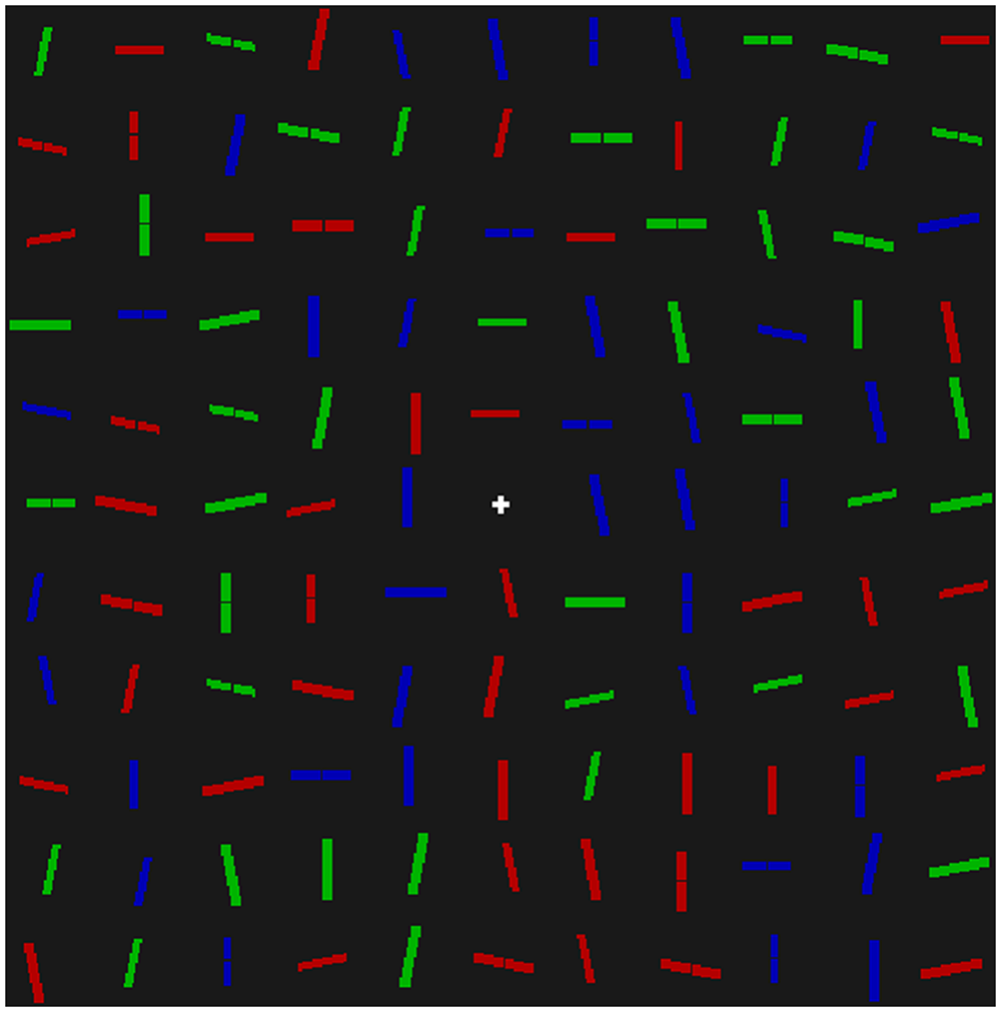
An example of the search displays used in Experiment 1. Displays consisted of 120 line segments of various orientations, colours and sizes. Participants were asked to search for the big red horizontal line segment, and to respond to the presence/absence of a notch in this target line.

The stimulus consisted of 120 lines, presented on an 11 x 11 grid, subtending a visual angle of 17° in height and width, as shown in Fig 1. Lines could be of two lengths: designated big (1 x .25°) or small (.8 x .2°); three colours: red (25.71 cd m^-2^), green (71.03 cd m^-2^), or blue (8.54 cd m^-2^); and six orientations: vertical, horizontal or ±12.5° from vertical and horizontal. The target was always a big red horizontal line, and this was unique, so that no distractor shared all three properties. The distance from the centres of two adjacent lines was on average 1.5°, but the centres were jittered at random by up to .2°. All lines had a 50% chance of being bisected by a 0.1° black “notch”. The background of the screen was black (0.01 cd m^-2^) and kept constant during the experiment.

**Fig 2:**
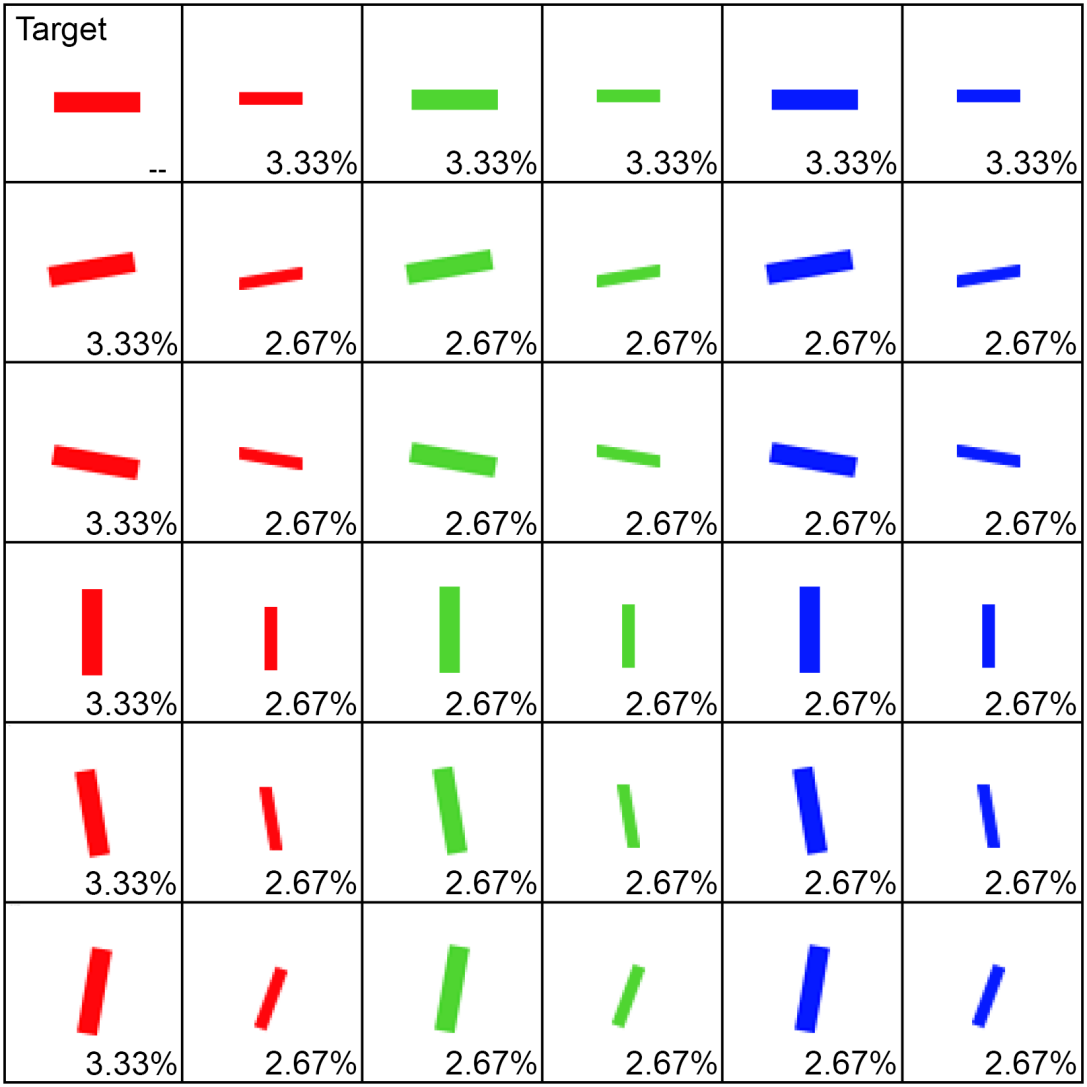
The full set of 36 possible combinations (3 colours, 6 orientations, 2 sizes) from which the 120 elements in the distractor sets were drawn. The big red horizontal line (top left) is the target, leaving a total of 35 possible distractors. Due to the removal of one possible combination for the target, the probability of each distractor being chosen to be in the distractor set was adjusted so that there would be an equal probability of a line being a certain colour, orientation or size. The adjusted probabilities are shown in the bottom right corner of each cell.

In the first block, 12 distractor sets were randomly generated (i.e., randomly assigning a colour, orientation and size to each distractor), according to the weightings in Fig 2. These weightings were set so that the distractors would have an equal chance to be any orientation, size or colour. The slight variation in weightings over distractors was necessary to counteract the effect of the unique target occupying one of the 36 possible feature combinations and therefore slightly biasing the algorithm. With these weightings, there is an equal chance that a distractor will be of any colour, orientation, or size. In subsequent blocks, the distractor sets were generated using a genetic algorithm, based on the participant’s performance in the first generation.

#### The Genetic Algorithm

**Fig 3:**
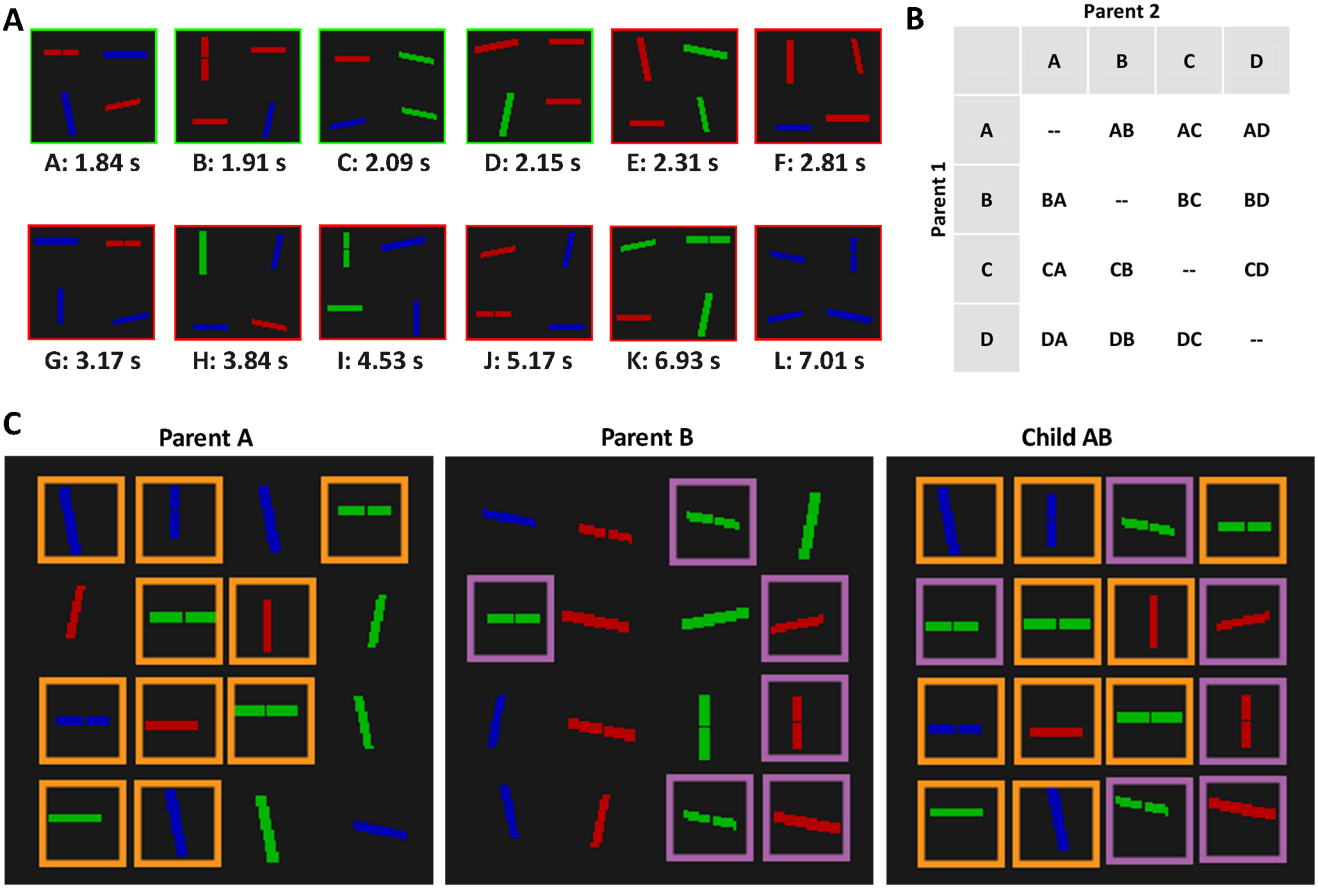
The genetic algorithm procedure used in Experiment 1. Panel A depicts the Testing and Selection phases of the procedure. The 12 unique distractor sets that participants searched through are labeled A to L and are ranked by the median reaction time based on 5 repetitions. Here, distractor sets A to D produced the fastest searches and are therefore selected to reproduce (indicated by the green boxes), while distractor sets E to L produced the slowest and are discarded (red boxes). Panel B depicts the start of the Crossover phase of the procedure. The 4 selected distractor sets are paired in all combinations, excluding repeats. Panel C depicts the rest of the Crossover phase, where lines from Parent A and Parent B are used to create Child AB.

The genetic algorithm was used to investigate visual search by manipulating the composition of, and thus the amount of competition in, the display. For the first block, participants performed the speeded search task on 60 displays, made up of 12 random distractor sets, each repeated 5 times, with the location of the target and distractors randomly determined on each trial. After the first block (i.e., the first generation), the fitness of each distractor set was computed by taking the median RT of the five displays that were created from the distractor set, with errors discarded and not analysed. Subsequently, based on a “survival of the fittest” principle, we selected the best four distractor sets from the set of 12 (i.e., those with the fastest median RTs) to create a new generation, a set of 12 evolved distractor sets created using a uniform crossover and mutation procedure. An example of these genetic algorithm processes is shown in Fig 3. The crossover procedure works by taking the four selected distractor sets and pairing them in all possible combinations, excluding repeats, as shown in Fig 3b. 70% of the lines in Parent A, and 30% of the lines in Parent B, are randomly selected, as represented by the orange and purple boxes in Fig 3C respectively. These lines are then combined to create Child AB. The 70–30 split helps to differentiate between Child AB and Child BA. Note that this crossover procedure is different to the one used in the Van der Burg et al., study [7], where a one or two-point crossover was used. In a uniform crossover, the child takes distractors from the parents, whereas in a one or two-point crossover, the child takes sections of distractors from the parents. A uniform crossover is slower to evolve in the early stages, but is better able to reject bad distractors, which may otherwise be sandwiched between good distractors.

Following the crossover procedure, a 4% mutation was applied to maintain diversity within a generation and avoid local minima. To do this, each line in each distractor set had a 4% chance of randomly changing into another feature combination according to the weights in Fig 2, such that on average, 4% of the lines in each generation were mutated. Note that this is also different from the Van der Burg et al., study [7], where there was a probability that a distractor chosen for mutation could mutate into the same feature combination, effectively lowering the mutation rate to 3.5%. The resulting 12 distractor sets formed the new generation of visual search stimuli for the next block, in which the participant searched through 60 displays, 5 from each of the distractor sets. This procedure was repeated until participants completed eight generations. Eight generations was chosen because Van der Burg, et al. [7], showed that most of the major changes occurred by the eighth generation in their study.

#### Procedure

Each trial began with the presentation of a white fixation cross (.5° in height and width; 100 cd m^-2^) at the centre of the screen for 500 ms. Subsequently, the search display was presented, and participants were instructed to search as quickly and accurately as possible for the big red horizontal line, by pressing the left or right arrow keys when a notch was absent or present on the target line, respectively. On 50% of the trials, the notch was present. After the participants’ response, the display became blank, and the next trial was initiated after 500 ms. Participants completed the search task for 480 displays per session, made up of 8 blocks (i.e., generations) of 12 distractor sets, each set creating 5 displays by shuffling the locations of the target and distractors. Participants had the opportunity to take a break every 30 displays. The experiment was preceded by a practice block of 60 trials (5 displays from 12 random distractor sets).

#### Results and Discussion

**Fig 4:**
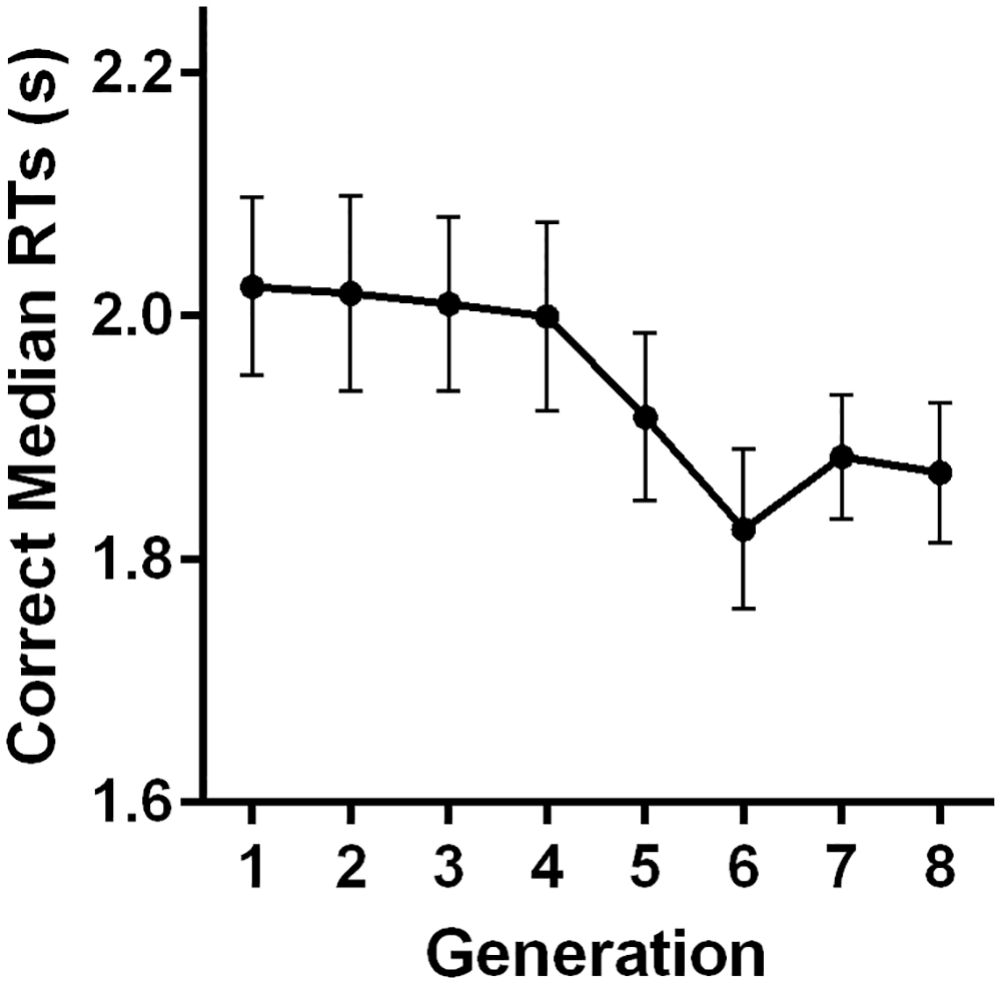
Correct median RTs across each generation of distractor sets, averaged over all participants. Error bars indicate ±1 within-subjects standard error of measurement [23].

To confirm that the genetic algorithm was successful in evolving the displays towards minimum RTs, we analysed the median RTs across each generation, as shown in Fig 4. The repeated-measures ANOVA showed that there was a significant generation effect, *F*(7,147) = 2.62, *p* = .014. Trend analyses found a significant linear trend, *F*(1,147) = 14.35, *p* = .001, but no other higher order trend, all *p*s > .204 indicating that the significant generation effect was due to the median RT decreasing over generations. The error rate was overall very low (1.64%) and therefore not further analysed.

From the decline in RTs over generations, it appears likely that the genetic algorithm was successful in changing the distractor sets to make participants faster at the visual search task. It is important to note though, that the RT data itself cannot determine whether the distractor sets evolved in a meaningful way, as other factors can also affect RT, such as practice and fatigue. While these effects are pronounced in comparisons of RT across generations, within a generation the effects are mitigated by the random order and repeated presentations. Since the genetic algorithm decides the evolution based on the RTs within a generation, the evolution is not affected by such effects.

**Fig 5:**
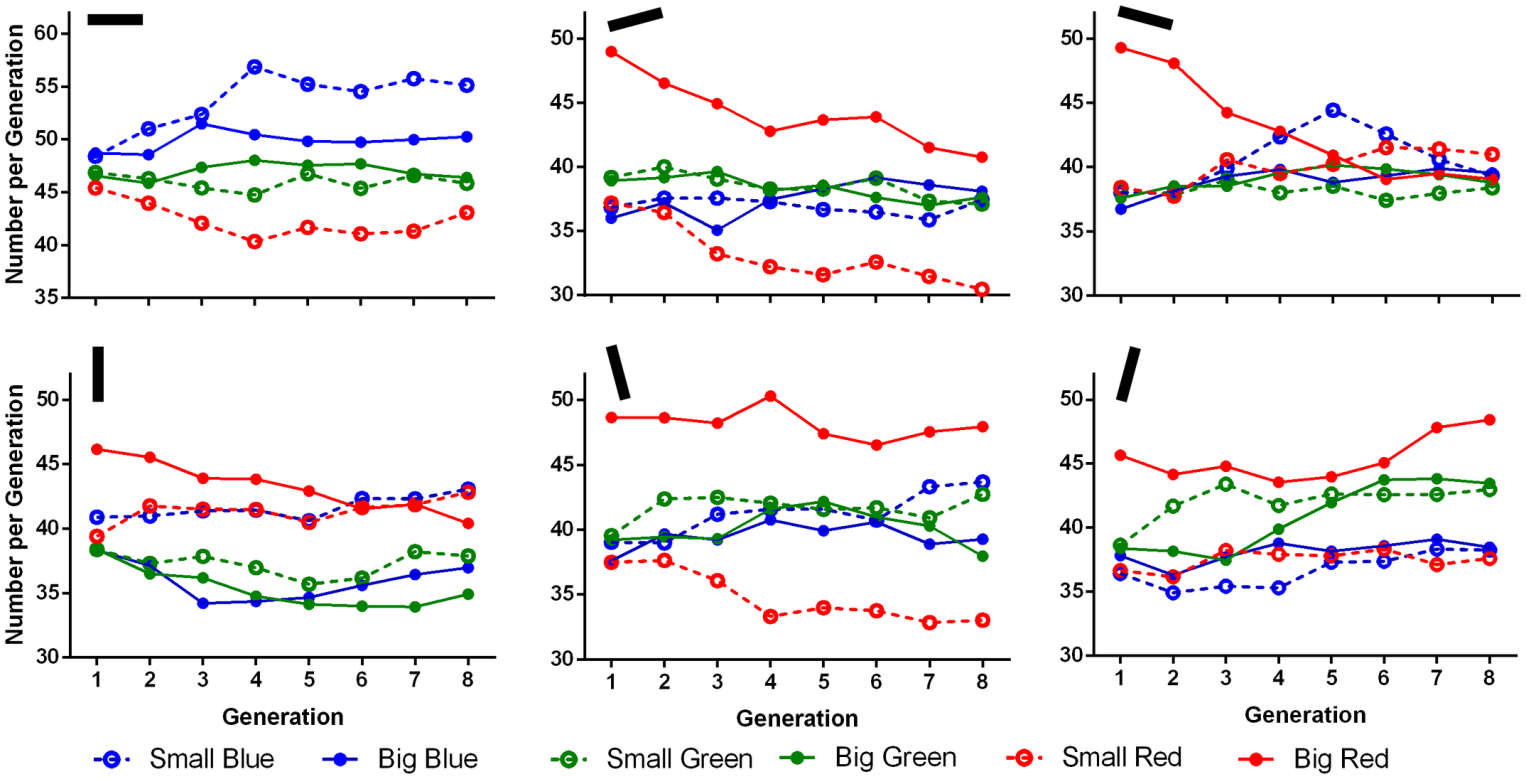
Results of Experiment 1 showing number of each distractor across generations, averaged across participants. Each distractor is represented as a line. Lines are arranged onto separate graphs based on their orientation, as marked indicated on the black bar on the top right of each graph. Within each graph, filled circles and unbroken lines represent big distractors, while unfilled circles and broken lines represent small distractors. The circles’ and lines’ colours represent the distractors colours.

Another way to determine whether the genetic algorithm was successful in evolving the distractor sets is to look at the changes that the genetic algorithm made across generations. If the decrement in RT over generations was merely due to a practice effect, then no systematic distractor changes over generation would have been expected. As is clear from Fig 5, the biggest notable changes were among the red distractors, with many of them decreasing to some degree. Vertical distractors were also notable for their lack of change.

Before analysing the frequencies with a statistical procedure, it is important to note that the data is compositional, i.e., the sum of all distractors must invariably add up to the total number of lines in the display. Because of this, there is perfect multicollinearity; the quantity of any distractor can be perfectly predicted by the others, creating artefactual correlations between the quantities of the distractors. For example, an increase in quantity of any one distractor invariably causes a decrease in the rest, creating a positive correlation among them. Since we are not interested in the absolute quantities of each distractor, but the quantities of the distractors relative to each other, the absolute quantities can be transformed using log-ratios, which has been shown to eliminate artefactual correlations [24]. However, log-ratio transforms cannot handle zero values, so where zero values appear, we have applied a multiplicative zero correction to the data [25]. To analyse the distractors, we have used an isometric log-ratio transform. The transformed data was analysed with a two-way repeated-measures ANOVA with Greenhouse-Geisser correction, which found a significant effect of distractor type, *F*(9.19,340.00) = 4.97, *p* < .001, indicating that there were more of some distractors than others. This is likely a reflection of the adjusted baseline of red items. The effect of generation was not significant *F*(2.75,101.83) = 2.17, *p* = .102, which is unsurprising because the number of distractors per generation was fixed. The interaction effect between distractor and generation was significant *F*(14.16,524.05) = 2.19, *p* = .007, indicating that the quantity of the distractors changed at different rates across generation.

**Fig 6:**
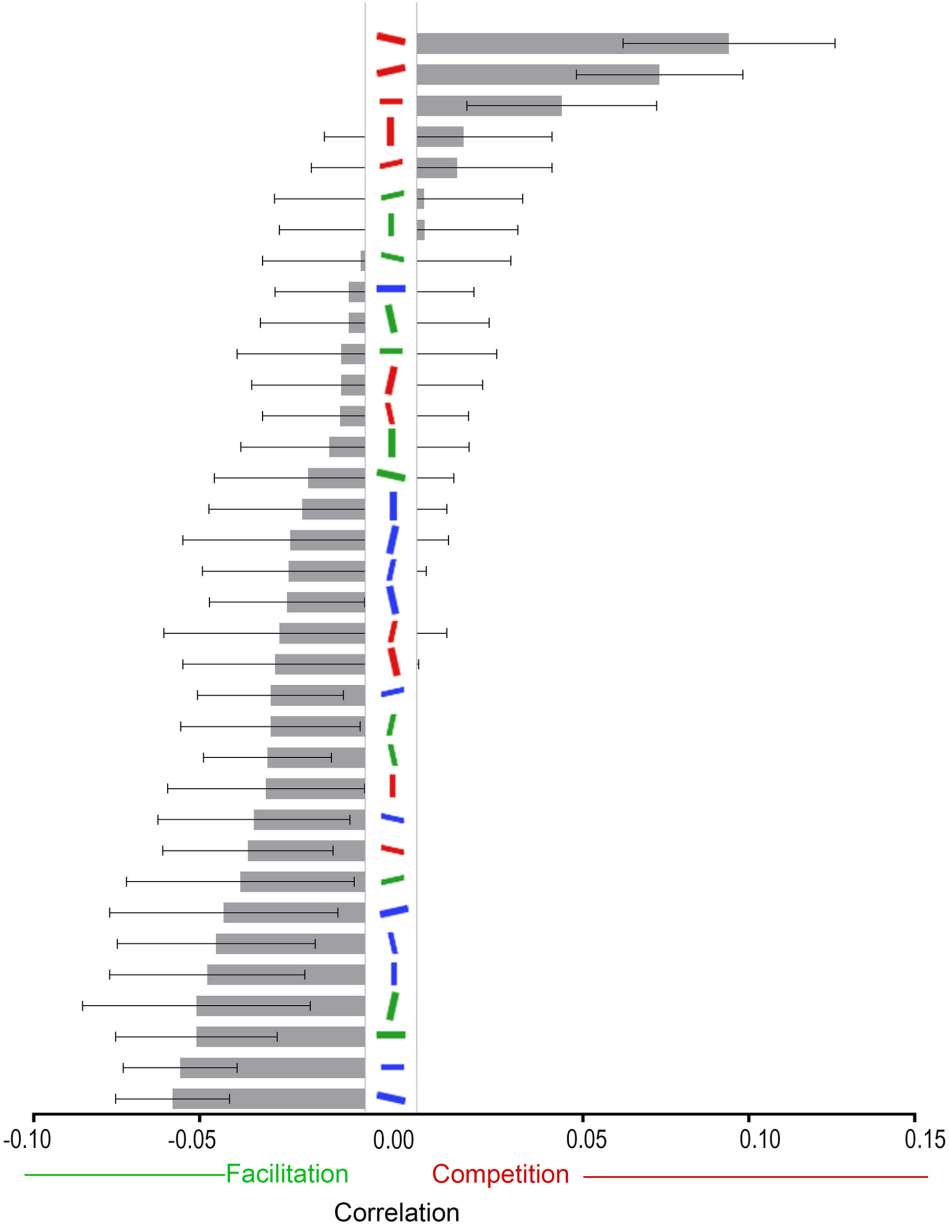
Correlations from Experiment 1 between distractor type and RTs, averaged across participants. Error bars represent ±1 within-subjects standard error of measurement [23]. * Indicates that the correlation is statistically significant at the α = .05 level after Bonferroni correction. + Indicates that the correlation is only statistically significant without Bonferroni correction.

Despite this significant interaction, there is no guarantee that a change in quantity of a certain distractor is meaningful, i.e., that the change in quantity reduced the reaction times and vice versa. So instead of running contrasts to see the changes in the absolute frequency, we correlated the quantity of each distractor in each distractor set across all generations with the median RT of the displays created by that distractor set, for each participant. By doing this, we analyse only the changes in the quantity of distractors that relate to changes in reaction time. Again, because of the compositional nature of the quantity data, we had to apply a log-ratio transform, with a multiplicative zero correction [24, 25], to correct for erroneous correlations between the distractors. In order to allow for a one-to-one correspondence between the transformed variable and the raw value, this time we used a centred log-ratio transform. The correlations were then averaged across participants, with the results shown in Fig 6. Note that a positive correlation for a specific feature combination signifies that the distractor competes with the target, and a negative correlation indicates that the distractor facilitates search.

A repeated-measures ANOVA with Greenhouse-Geisser correction on the 35 correlations showed that there were statistically significant differences between the correlations, *F*(11.43,240.10) = 2.83, *p* = .001. Bonferroni-corrected *t* tests comparing those correlations against zero (see S1 Table) showed that the big red 12.5° from horizontal and big red -12.5° from horizontal distractors were significantly positively correlated with RT, indicating that the presence of these distractors was detrimental to visual search. The small blue horizontal and big blue 12.5° from horizontal were also significantly correlated, but negatively, indicating that the presence of these distractors facilitated visual search. All other correlations were not significantly different from zero.

From the correlations between RTs and distractor type, we can conclude that the distractors most detrimental to visual search were the big red 12.5° from horizontals and the big red -12.5° from horizontals, and that the most facilitatory were the big blue 12.5° from horizontals and the small blue horizontals. The small red horizontal distractor was the only other distractor that might be positively correlated (*p* = .011), but Bonferroni correction meant it did not reach the corrected level of statistical significance. Qualitatively speaking, the red distractors occupy the positive end of Fig 6, while non-red horizontal-ish distractors mostly occupy the negative end. This latter effect of more distractors sharing the target’s orientation leading to faster RTs replicates what Van der Burg et al., [7] found, that horizontal lines seem to facilitate visual search, despite the target also being horizontal.

Before considering the theoretical implications of the results, it is important to note that the genetic algorithm, or at least the way we have applied it here, has certain limitations that should be noted when interpreting these results. The first is that each display must have a fixed number of elements, which means that when one specific distractor type disappears another must appear to take its place. Thus, in principle, the increase in small blue horizontal distractors may simply be an artefact due to the decrease in red horizontal-ish, distractors, or vice versa. This seems unlikely in the present experiment given the large number of possible feature combinations; however, we cannot dismiss this possibility entirely. Nonetheless we are not inclined to believe that the decrease in red horizontal-ish, distractors causes an artefactual increase in small blue horizontal distractors because any associated increase should be equally distributed across all the remaining feature combinations, especially since the genetic algorithms were run independently for each of the 22 participants, each starting with 12 unique initial distractor sets.

Another possible issue was that compared to the Van der Burg et al., [7] study, we have found a relatively low amount of improvement in the RTs and low correlations. A possible reason for this is the increased search space of this experiment. Our displays contained 119 distractor lines, each with a possible 35 values, leading to a total of 35^119^ distractor sets for the genetic algorithm to search through. In comparison, Experiment 1 of the Van der Burg et al., study contained 8^72^ possible distractor sets for it to search through. We do not believe the low magnitude of our results invalidates our results, as later experiments will demonstrate, but researchers wanting to use the method in the future should be aware that we may be approaching the limit for how big a problem space our application of the genetic algorithm can handle. There are ways to extend this limit, the most obvious way being to increase the data available to the genetic algorithm, i.e., increasing the number of generations or increasing the number of children per generation. However, this is limited by the time each participant can put into the experiment. Another way to extend the limit is to make the contribution of each distractor consistent, by fixing the location of each distractor between presentations, e.g. Experiment 2 of the Van der Burg et al., study. This improves the accuracy of the fitness values, allowing for more efficient changes every generation. Another possibility is to allow the genetic algorithm better access to the possible solutions. Given that there are only two parents for every child, only 2 of the 35 possible feature combinations could be accessed by that pair. If it makes sense to do so, a different encoding scheme could allow a pair of parents to access many more distractors combinations, e.g., in another of our studies [26], the distractors were encoded using a Gray code, allowing a pair to access on average half of the possible feature combinations in any pair.

Another issue with genetic algorithms is the possibility that they evolve towards a local minimum, not the global minimum. A local minimum can occur in a multi-dimensional genetic algorithm, such as the one we used here. To illustrate, imagine that all the horizontal genes disappeared early because they were on average unfit, but in reality, certain coloured horizontals were highly fit. If no horizontal genes remain after early generations, the genetic algorithm will not be able to find the fit horizontal-colour combinations and will instead settle in a local minimum. Again, while we cannot dismiss this possibility, we do not believe this was the case. Firstly, we have incorporated a relatively large mutation factor (4%) into our genetic algorithm, so that any gene that disappears has a decent chance of reappearing every generation in one of the 12 distractor sets. Secondly, all participants started with a different initial set of distractors in the first generation, making it very unlikely that all participants evolved to the same local minima. Thirdly, although our displays contained three dimensions (3 colours, 6 orientations, 2 sizes), we have operationalised it as a single dimension (35 distractor possibilities). Thus, each combination of features was assessed by the genetic algorithm, not the feature dimensions, making it very unlikely that a whole dimension would disappear, especially given that the experiment consisted of only eight generations.

The other common way for a local minimum to occur is for the global minimum to stand in isolation. This occurs when any slight change to the features of the global minimum would lead to a disproportionately large drop in its fitness value. This would occur in visual search, for example, when the display is homogeneous. Any distractor could be classified as the fittest if all other distractors were also of the same type, as this would produce a 'pop-out' effect. However, as the point of the study was to explore heterogeneous displays, we are less concerned about these types of global minima problems. Furthermore, it would not have been possible for our genetic algorithm to include homogenous displays in the first place, given that the mutation factor enforces at least 4% heterogeneity.

Despite these concerns, the significant differences in the correlations suggest that the genetic algorithm found something meaningful in our results (see S1 Text for a proof of this). Given the low magnitude of the effects though, and the novelty of the method, we can treat these results simply as exploratory data, which can be confirmed using targeted, factorial designs. Factorial designs were previously not feasible due to the sheer number of possible test conditions, but the results from the genetic algorithm can be interpreted as finding test conditions of interest. Thus, Experiments 2 and 3 seek to confirm the results of our genetic algorithm using factorial designs to target certain test displays.

## Experiment 2

The first result that requires confirmation is the detrimental effect of red horizontal-ish distractors. To do this, we reused the same type of search display as in Experiment 1 (see Fig 1) but rigged the quantities of certain distractors in one of four ways. To confirm the results of the detrimental distractors in Experiment 1, we increased the number of big red 12.5° from horizontal and big red -12.5° from horizontal lines in the display. If increased numbers of big red ±12.5° distractors are detrimental, then search times for these displays should be slower than on control displays. Similarly, to see if the lack of a statistically significantly positive correlation between RT and the small red horizontal distractor was due to the conservative Bonferroni correction procedure, we increased the number of small red horizontal distractors. Again, if this were the case, we should see an increase in search times compared to controls. Two control displays were also tested, the first being randomised displays (the same as in the first generation of Experiment 1), which provided an absolute baseline. The second control display consisted of an increase in small red 12.5° from horizontal and small red -12.5° from horizontal lines, which provides a control for the case that the detrimental effect is merely driven by the general increase in red distractors.

### Materials and Method

#### Ethics Statement

Written consent was obtained from each participant prior to the experiments. The experiments were approved by the local ethics committee of the University of Sydney in accordance with the Declaration of Helsinki.

#### Participants

12 participants (9 female, mean age = 22.17, ranging from 18 to 33 years) took part in this experiment. All participants had normal or corrected-to-normal vision. All participants were naïve as to the purpose of the experiment.

#### Materials

**Fig 7:**
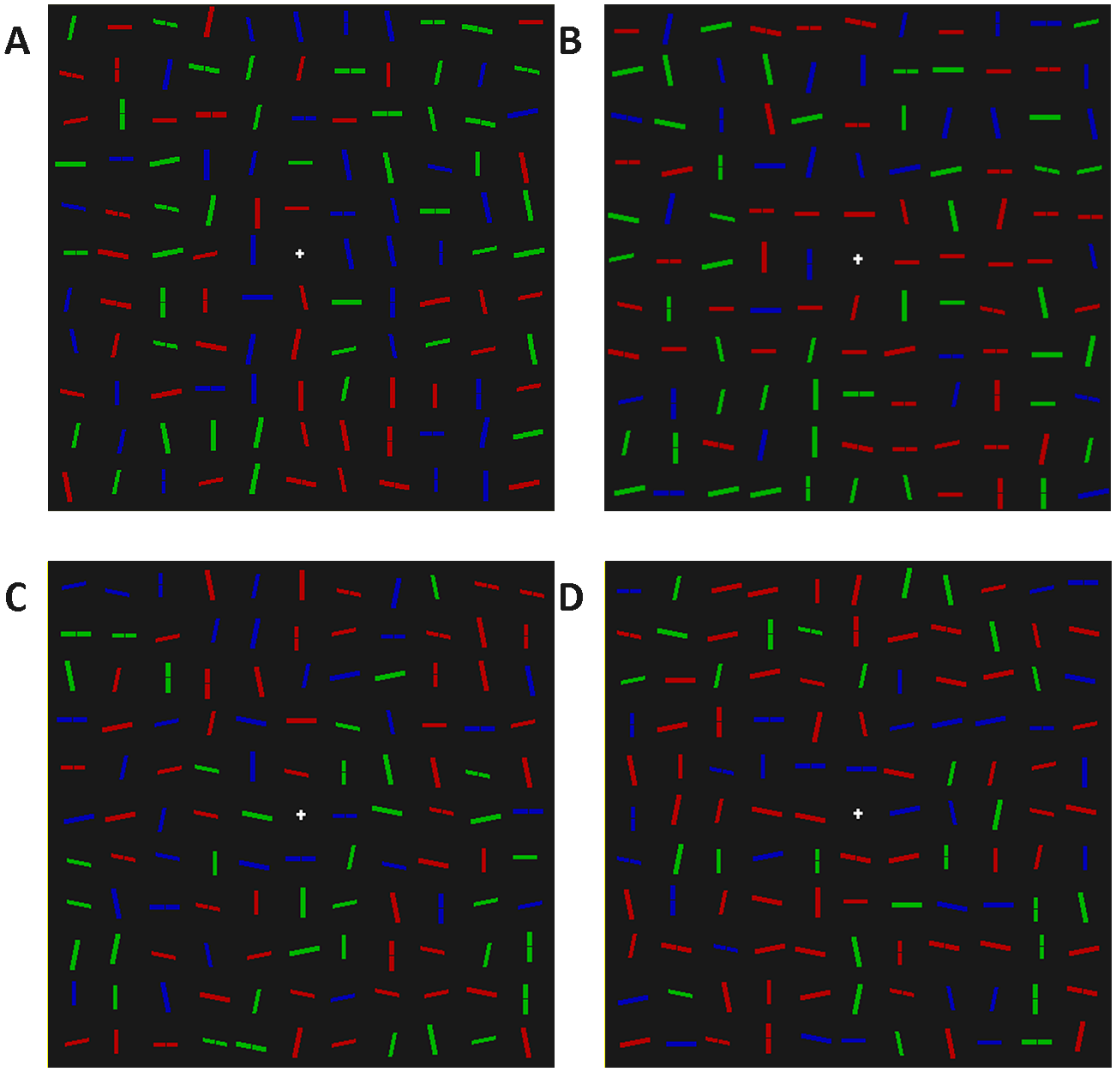
Example stimuli for the three non-random conditions of Experiment 2. Display A is the baseline control condition, created in the same way as in the first generation of Experiment 1. In display B, the probability of small red, horizontal distractors has been increased. In display C, the probability of small red, 12.5° from horizontal and small, red -12.5° from horizontal distractors has been increased. In display D, the probability of big red, 12.5° from horizontal and small red -12.5° from horizontal distractors has been increased.

The visual search display was the same as in Experiment 1, with the following exceptions. The composition of the display was not determined by a genetic algorithm, but chosen from one of four display compositions. Two of the compositions were control conditions: i) Same as the initial (random) displays in Experiment 1, and ii) 10% were small red 12.5° from horizontal, 10% were small red -12.5° from horizontal, and the remaining 80% were randomly chosen from the rest. The other two display compositions were experimental conditions: iii) 20% of lines were small red horizontal, the remaining 80% were randomly chosen from the rest, and iv) 10% were big red 12.5° from horizontal, 10% were big red -12.5° from horizontal, and the remaining 80% were randomly chosen from the rest. Examples of the displays can be found in Fig 7.

#### Procedure

Participants searched 120 displays in total. The four distractor conditions were displayed in a random order, i.e., unblocked. All participants did 40 practice search displays before starting the experiment.

#### Results and Discussion

**Fig 8:**
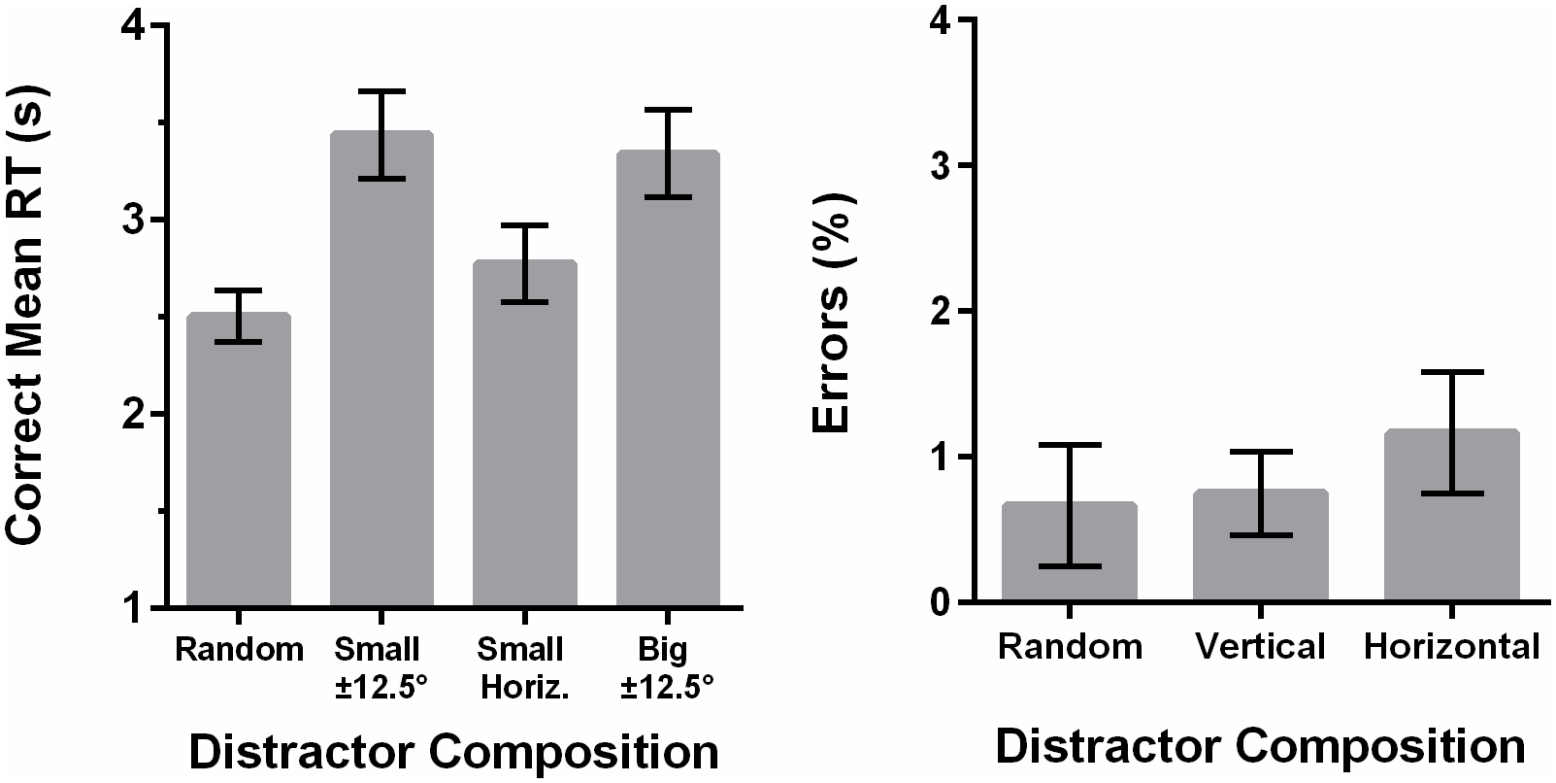
Data from Experiment 2 showing correct mean RTs as a function of the display composition. Error bars represent ±1 within-subjects standard error of measurement [23]. The x-axis indicates which of the red distractors were systematically increased in that condition. For example, small ±12.5° indicates that 20% of the display was fixed as small red 12.5° from horizontal and small red -12.5° from horizontal lines.

Incorrect trials were discarded, leading to a loss of 2.71% of the trials. RTs that were more than 3 SDs away from the mean of that condition were deemed as outliers and discarded, leading to a loss of an additional 1.11% of the total trials. A repeated-measures ANOVA on the errors revealed no significant effect of display type, *F*(3,33) = .26, *p* = .859. Mean RTs for each display composition are shown in Fig 8.

A repeated-measures ANOVA on the mean RTs revealed a significant display composition effect, *F(*3,33) = 10.16, *p* < .001. We also performed contrasts testing the three other display types against the randomised control. Participants were slower on the 20% small red horizontal, *t*(11) = -5.34, *p* < .001) and 20% big red ±12.5° from horizontal displays, *t(*11) = -4.69, *p* = .001, i.e., big red ±12.5° from horizontal distractors were found to hinder visual search performance. The 20% small red ±12.5° from horizontal displays were found to be not statistically different from control, *t(*11) = -1.80, *p* = .100, indicating that these distractors were not noticeably detrimental to visual search. This also indicates that the general increase in RTs is not due to the increased number of red items, but that orientation specifically played a crucial role. Overall, these results provide support for the interpretation that the results from Experiment 1 were neither merely an artefact of the genetic algorithm, nor invalidated by the limitations discussed earlier.

## Experiment 3

The other result from Experiment 1 that requires confirmation is the facilitatory effects of the non-red horizontal distractors. Once again, we reused the same type of search display as in Experiment 1 but altered the display composition in one of three ways. In the experimental display, we increased the number of small or big, green or blue, horizontal lines. Two control displays were also used. The first was a randomised display as in the first generation of Experiment 1. The second was a display with increased numbers of small or big, green or blue, vertical lines. If the results from Experiment 1 are not due to an artefact, and there is a search advantage to non-red horizontal lines, then we would expect the fastest RTs to be found in the experimental condition. We would also expect that the control display with the increased number of non-red vertical lines to have lower RTs than the randomised control display, as there is a general decrease in the number of red items, but importantly any difference between this control display (increased verticals) versus the experimental condition (increased horizontals) can be attributed to the orientation, and not to the decrease of red items in the display.

### Materials and Method

#### Ethics Statement

Written consent was obtained from each participant prior to the experiments. The experiments were approved by the local ethics committee of the University of Sydney in accordance with the Declaration of Helsinki.

#### Participants

12 participants (8 female, mean age = 21.42, ranging from 19 to 29) took part in this experiment. All participants had normal or corrected-to-normal vision. All participants were naïve as to the purpose of the experiment.

#### Materials

**Fig 9:**
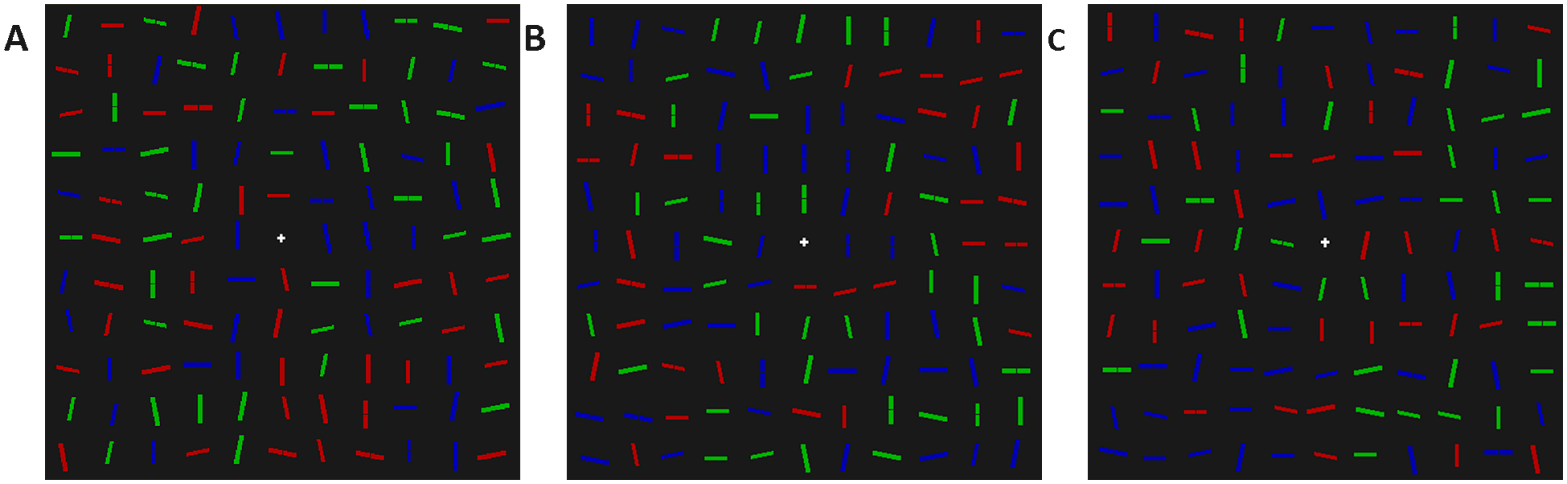
Example stimuli for the two non-random conditions of Experiment 3. A) The baseline control condition, created in the same way as in the first generation of Experiment 1. B) Display composition in which the probability of non-red vertical distractors has been increased. C) Display composition in which the probability of non-red horizontal distractors has been increased.

The visual search display was the same as in Experiment 2, with the following exceptions. There were three display compositions, two of which were control conditions: i) Same as the initial (random) displays in Experiment 1; ii) 20% of lines were small or big, green or blue, vertical lines, with the remaining 80% randomly chosen from the rest of the distractor categories; and a third, which was an experimental condition: 20% of lines were small or big, green or blue, horizontal lines, and the remaining 80% were randomly chosen from the rest of the distractor categories. Examples of the displays can be found in Fig 9.

#### Procedure

Participants searched 120 displays in total. The three distractor conditions were displayed in a random order, i.e., unblocked. All participants did 30 practice search displays before starting the experiment.

#### Results and Discussion

**Fig 10:**
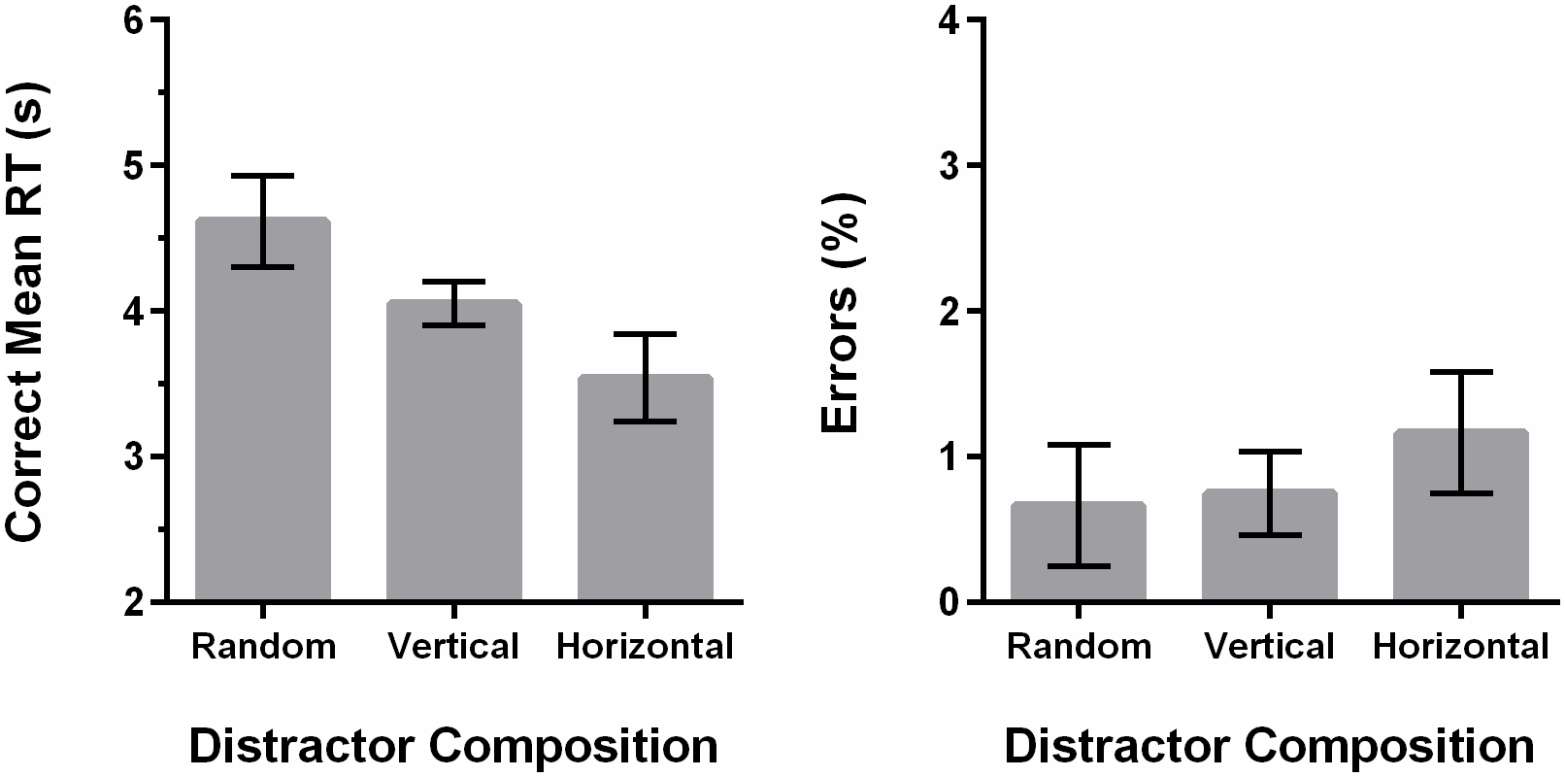
Data from Experiment 3 showing correct mean RTs as a function of the display composition. Error bars represent ±1 within-subjects standard error of measurement [23]. X-axis labels indicate the non-red distractors that were increased in each condition. For example, vertical indicates that 20% of the distractors in that display were fixed as non-red vertical distractors.

Incorrect trials were again discarded, leading to a loss of 2.16% of the trials. RTs that were more than 3 SDs away from the mean of that condition were deemed as outliers and discarded, leading to a loss of an additional 1.38% of the total trials. A repeated-measures ANOVA on the mean error rate yielded no significant display composition effect, *F*(2,22) = 1.00, *p* = .384. Mean correct RTs for each display type are shown in Fig 10.

A repeated-measures ANOVA with Greenhouse-Geisser correction on the RTs revealed a statistically significant effect of display type, *F(*1.36,14.96) = 8.30, *p* = .007. Pairwise contrasts revealed that participants were faster in the 20% non-red vertical condition, *t*(11) = 2.55, *p* = .027, and the non-red horizontal condition, *t*(11) = 3.28, *p* = .007) than the randomised control condition. Importantly, participants were faster in the non-red horizontal condition than the non-red vertical condition, *t*(11) = 2.74, *p* = .019, indicating that observers are faster at searching for a big red horizontal target when there are more non-red horizontal-ish distractors in the display. This effect does not appear to be purely due to the reduced number of red distractors in the display, as this facilitatory effect is bigger when the non-red distractors are horizontal than when they are vertical. Overall, the results confirm our qualitative interpretation of the results in Experiment 1. This facilitation effect has been discovered before (Van der Burg et al., 2015), but here we show that this is not due to a systematic artefact of the genetic algorithm. However, such an effect is puzzling, as it defies our current understanding of visual search. Colour, size and orientation are all known to be “guiding features” of visual search [2] and the similarity between the target and distractor of such features is known to make visual search slower [3]. More specifically, in visual search with three feature dimensions, the number of shared features has been shown to affect search rates [6, 27]. Thus, in our displays, the non-red horizontal distractors, which share one or two features (orientation and size) with the target, should have been more detrimental to visual search times than the non-red vertical distractors, which share only one feature (size) or none.

The inability of current models to explain our results raises the issue of why this facilitation effect has not been noticed before. The most obvious possibility is that the effect is specific to the displays used here. Studies that have investigated search with more than two feature dimensions have typically been compromised for ease of analysis. For example, in their most heterogeneous display, Nordfang and Wolfe [6] investigated visual search involving six feature dimensions, but only six feature combinations were ever present in a single trial and a maximum set size of 36. Similarly, Weidner and Müller [28] used a search display with three feature dimensions, but only five feature combinations were ever present in one trial, with a set size of 25. In both cases, because of the use of a small subset of the possible feature combinations, the distractors then have multiple copies and thus distractor-distractor similarity is increased [3]. In contrast, our maximum of 35 feature combinations with a set size of 120 makes for a visual search display with more heterogeneity, both compared to the target and among the distractors themselves. It is entirely possible that participants respond differently to highly heterogeneous displays than they do to more homogenous ones. Indeed, Nordfang and Wolfe also had a more heterogeneous display where all 26 possible feature combinations from three feature dimensions were used, and found that participants were both slower and less efficient than when they used only a subset of the possibilities.

There are also theoretical reasons to suspect that heterogeneity is changing search behaviour. Heterogeneity appears to have a non-linear relationship with reaction times [3, 6], which suggests that not only is searching heterogeneous displays more difficult, but the process of searching through them may be different. One reason for the non-linearity may be that people search within a subset of distractors and ignore the others [29, 30]. Another reason may be that multiple, similar distractors may be being search as a group [31], decreasing the number of “items” that needs to be searched through. Regardless, the finding of non-linearity in heterogeneous visual search makes us suspect that the facilitatory effect of the non-red horizontal-ish distractors may be restricted to heterogeneous displays.

## Experiment 4

In Experiment 4 we manipulate the heterogeneity of the displays to examine whether the facilitation effect of the non-red horizontal distractors on search for a red horizontal target is contingent upon the heterogeneity of the display. While there are multiple ways to manipulate heterogeneity, we have chosen to do this by fixing the number of non-red horizontal-ish distractors to 20%, 50% or 80% of the display. These proportions were selected so that the most homogenous condition would be most similar to experiments typically done in visual search, i.e. targets are found quickly due to a bottom-up signal, while keeping the overall structure of the stimulus the same across heterogeneity levels. A total of four baseline conditions were created: three respective control conditions by fixing the non-red vertical distractors by the same amount, and one which was the randomised displays as in the first generation of Experiment 1. If the facilitation effect of the non-red horizontal distractors is reliant on the heterogeneity of the display, then we expect that the difference in RT between the displays with increased horizontal distractors and displays with increased vertical distractors (i.e., the control condition) to be more pronounced as heterogeneity increases.

### Materials and Method

#### Ethics Statement

Written consent was obtained from each participant prior to the experiments. The experiments were approved by the local ethics committee of the University of Sydney in accordance with the Declaration of Helsinki.

#### Participants

12 participants (8 female, mean age = 21.42, ranging from 19 to 29) took part in this experiment. All participants had normal or corrected-to-normal vision. All participants were naïve as to the purpose of the experiment.

#### Materials

**Fig 11:**
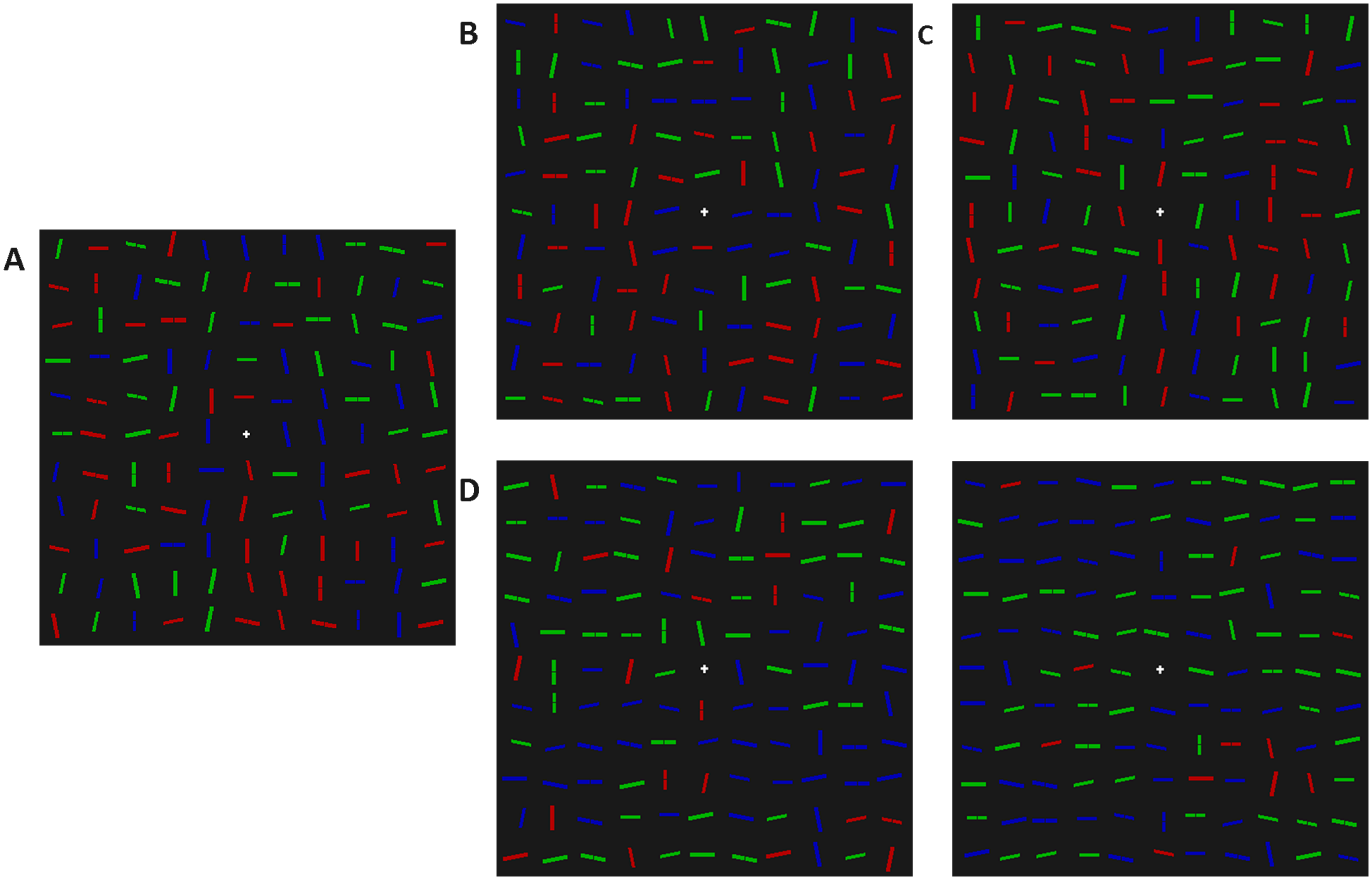
Example stimuli for the three non-random conditions of Experiment 4. Display A is the baseline control condition, created in the same way as the first generation in Experiment 1. In display B, 20% of the distractors fixed as non-red horizontal-ish distractors. In display C, 20% of the distractors fixed as non-red vertical-ish distractors. In display D, 50% of the distractors fixed as non-red horizontal-ish distractors. In display E, 80% of the distractors fixed as non-red horizontal-ish distractors.

The visual search display was the same as in Experiment 3, with the following exceptions. There were seven display compositions in total. The three heterogeneity conditions were created by fixing the number of non-red, horizontal, non-red 12.5° from horizontal and non-red -12.5° from horizontal distractors as 20, 50 or 80% of the display, and selecting the rest of the distractors at random from the weighting in Fig 1. Three control conditions were created by doing the same thing, but for non-red vertical, non-red 12.5° from vertical and non-red -12.5° from vertical distractors. A fully randomised condition was also included, which was the same as the starting displays in Experiment 1. Fig 11 shows an example of the critical conditions.

#### Procedure

Participants searched 280 displays in total. The seven distractor conditions were displayed in a random order. All participants did 42 practice trials before starting the experiment.

#### Results and Discussion

**Fig 12:**
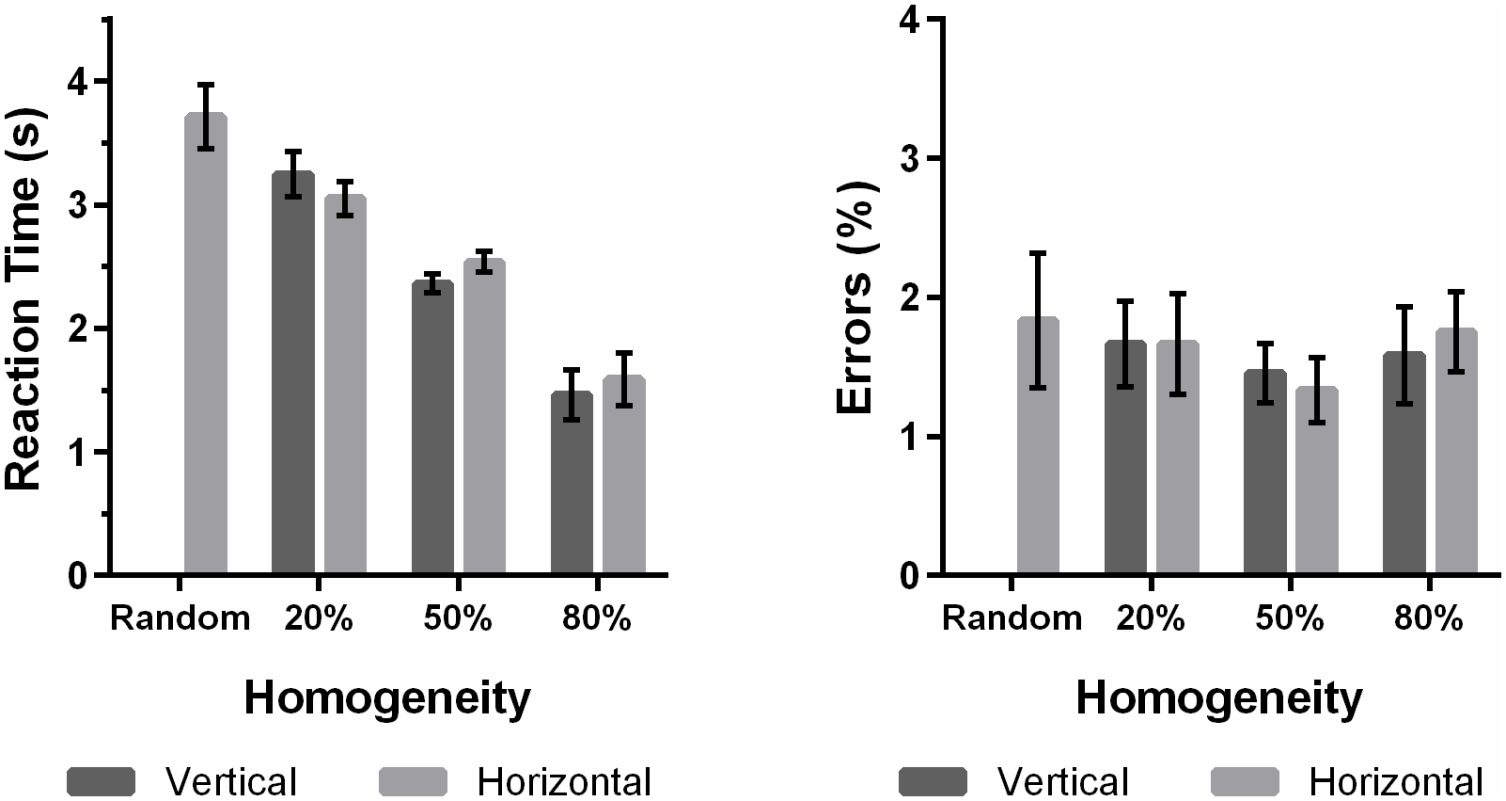
Data from Experiment 4 showing correct mean RTs as a function of the display composition. Error bars represent ±1 within-subjects standard error of measurement [23]. “Vertical” denotes displays in which the non-red vertical-ish lines were increased. “Horizontal” denotes displays in which the non-red horizontal-ish lines were increased.

Incorrect trials were discarded, leading to a loss of 2.11% of the trials. RTs that were more than 3 SDs away from the mean of that condition were deemed as outliers and discarded, leading to a loss of an additional 1.92% of the trials. A repeated-measures ANOVA on the errors revealed no statistically significant effect of the orientation manipulation, *F*(1,23) = .01, *p* = .922, or homogeneity, *F*(1.59,36.45) = 1.09, *p* = .335. The interaction effect was also not significant, *F*(2,46) = .24, *p* = .770. Mean correct RTs for each display type are shown in Fig 12.

The six experimental conditions were analysed with a 3 x 2 repeated-measures ANOVA with Greenhouse-Geisser correction, which indicated a significant main effect of homogeneity, *F*(1.18,27.11) = 49.21, *p* < .001, indicating RTs decreased with increasing homogeneity. The main effect of the orientation manipulation was not significant, *F*(1,23) = 1.06, *p* = .315. The interaction effect was significant, *F*(1.57,36.06) = 9.46, *p* = .001. To understand the interaction, we ran three orthogonal contrasts, all of which were significant. Within the 20% homogeneity condition, RTs for displays with increased non-red vertical-ish distractors were greater than for displays with increased non-red horizontal-ish distractors, *F*(1,46) = 4.67, *p* = .041, replicating the results from Experiment 1 and 3. This pattern was reversed in the 50% and 80% homogeneity conditions, *F*(1,46) = 9.60, *p* = .005 and *F*(1,46) = 13.89, *p* = .001 respectively. This reversal in the pattern confirms our suspicion that the facilitatory effect of the non-red horizontal-ish distractors is only present in heterogeneous displays. It also shows a possible limitation to the genetic algorithm as a technique. Given enough time, the displays may have evolved into the ones seen in the 50% and 80% homogeneous conditions, but the limited amount of generations we performed means that the algorithm only searched within a limited space of the possible displays. Of course, this is less of a problem if one is interested in heterogeneity, as we are.

However, the results provide an explanation as to why this facilitatory effect has not been noticed in previous studies, as most other studies use relatively homogeneous displays. Of course, the exact definition of heterogeneity is unclear, but from the results of this experiment, it should be uncontroversial to make such a claim. The 50% condition represents what would be classified as “heterogeneous” in a typical visual search study, with each of the 23 unmanipulated distractor elements appearing on average 1.6 times in each display. However, the facilitation effect was not noticed in this condition.

## General Discussion

This study has investigated how we perform visual search on heterogeneous displays. Experiment 1 used a genetic algorithm to manipulate the stimulus, allowing us to infer what effect each type of distractor had on visual search performance. Experiments 2 and 3 have shown that the results were not jeopardised by the issues raised in the Discussion of Experiment 1 by replicating the results using a conventional, factorial design. Overall, there are two key findings from these experiments. The first is that the red horizontal-ish distractors are detrimental to visual search when the target is also a red horizontal line. However, this relationship is dependent also on the size of the distractor. A distractor can be 12.5° off horizontal or differ in size from the target, and still be detrimental to visual search. However, when it is both 12.5° from horizontal and of a different size, then the detriment decreases to the point of non-significance. This finding can be explained by any model of visual search that incorporates partial bottom-up activations within a given feature dimension. For example, a signal detection theory based model of visual search (e.g., [19]) explains the results by assessing each distractor’s similarity to the target in all relevant dimensions.

Our second finding is that non-red horizontal-ish distractors are facilitatory when the target is red and horizontal ([see also [7]). This finding is counterintuitive as there should be no reason to attend to a green or blue line. Given that the display was heterogeneous, no bottom-up attentional slip should have occurred, i.e., attention should not have been drawn away from the target and towards a task-irrelevant distractor [32]. Furthermore, pre-attentive processing should have diverted attention away from green and blue lines as they did not share colours with the target. Even if the shared orientation drew top-down attention to them it should lead to increased RTs as they are not the target. In other words, participants should not have attended to the non-red horizontal distractors and there are no advantages to doing so. The difficulty in explaining this is reflected in how some models of visual search fail to explain it without additions. For example, guided search [33] would predict that non-red horizontal-ish distractors would receive some activation from the orientation dimension, and thus be more detrimental than the non-red vertical-ish distractors, which do not receive this activation. Similarly, a model based on signal detection theory [19] would predict the same, as the shared orientation means a higher activation for the non-red horizontal-ish distractors than the non-red vertical-ish ones.

The finding that a distractor that shares a feature with the target can facilitate visual search has not, and likely could not, have been found through an investigation of the simple visual search displays that are typically used. Simple displays are used because they provide a high degree of control over aspects of the stimulus, allowing the researcher to be confident that any effect found is due to their theorised concept. However, due to this high degree of control, it is difficult to identify processes that were not relevant to searching those simple displays. This leaves theories that were created purely from results of experiments using simple displays unable to fully explain search involving complex stimuli. This often manifests as a nonlinear increase in RT (e.g., [5, 6]). The facilitation effect described in this study is another example of such nonlinearity, but also suggests that the nonlinearities may be attributed to yet undiscovered effects.

The existence of effects only noticed in search in complex displays is especially important, as it has implications for the current movement towards the study of search in realistic scenes. Studies on realistic scenes, as the most complex (yet natural) of stimuli, often account for as much of the variance in search performance as possible using theories that apply to simple stimuli, and then propose extra factors needed to explain the differences. For example, guided search seems applicable to search in a household scene [34], but requires the addition of a semantic knowledge component. Similarly, salience models predict eye-movements well for target acquisition [35], but require top-down knowledge of the target. However, these extra factors may not be “extra” at all. They may also play a small, and thus undiscovered, role in search for simple stimuli too, and thus need to be incorporated into the theories, instead of acting as an extra factor.

The use of predominantly simple stimuli to create theories on visual search as a whole is understandable. Complex stimuli, by definition, consist of so many variables that it is difficult to determine what the additional process needed to explain search in complex stimuli is. Even if we were able to identify a variable of interest, the study of complex stimuli has another problem: the lack of precise control over the stimulus. Because of the many features that make up a complex visual search stimulus, one cannot be sure that a manipulation affects the desired concept and nothing else. For example, Experiment 4 was designed with the hypothesis that the facilitation effect required a heterogeneous display in order to work. Given that we only found this effect with the most heterogeneous of our displays, it would seem reasonable to identify heterogeneity as necessary. However, our heterogeneity manipulation could have systematically affected other features of the display that covary with heterogeneity, such as display complexity or effective set size. These were not controlled for, but could have conceivably had an effect. For example, the number of types of distractors in a 20% homogeneous display was on average more than in an 80% homogeneous display, increasing search times in the 20% displays [6]. Similarly, there is evidence that people search only within a subset of items [29, 30], meaning the effective set size, the items that people actually search through, would inevitably be bigger in the 20% homogeneous condition than any other, increasing search times.

Despite these other possibilities, we still feel that it is likely for heterogeneity to be the necessary condition for the facilitation effect to occur. Heterogeneity has long been known to affect visual search, with both target-distractor and distractor-distractor similarity affecting search times [3]. Furthermore, the effect of heterogeneity is thought to produce non-linear changes to search slope [6, 29] although this is difficult to argue, as it is hard to know if heterogeneity manipulations are truly linear. It has been argued that search in heterogeneous conditions is still serial, and thus linear, after accounting for similar distractors being grouped and dismissed for not being the target together [31]. However, this does not appear to be the case here, as grouping should have a bigger effect on the increased non-red verticals, allowing for all of such distractors to be dismissed at once.

In fact, the root of the difficulty in explaining the finding that non-red horizontal distractors facilitate the search for a red, horizontal target is that the sharing of features between target and distractor is detrimental to visual search [3, 6, 27]. Given the strength of the evidence behind the search detriment of target-distractor similarity, it is likely that there is a third, unknown effect in our visual search task that is triggered by or only noticeable in heterogeneous conditions. Going through the framework of Awh, Belopolshy and Theeuwes [36], this unknown effect is unlikely to due to physical salience, as there is practically no difference in the bottom-up signal between the two 20% homogeneous displays. If there were a difference, it would be in the opposite direction, i.e., an increased number of vertical-ish distractors should make it easier to create either a subset of horizontal items to search through [29, 30], or groups of vertical items to reject [31]. The task is also the same each time, which rules out the possibility of a goal-driven effect. This leaves the possibility of a selection history-based effect, but it is unknown how such a process could cause this. Various known effects, such as knowledge of the target [37], inter-trial priming [38] and contextual cueing [39] are controlled for through the randomisation of both the elements and the order of the displays in the experiment.

Another possibility is an interaction of the aforementioned effects. While such interactions are ill-defined in the literature, two possibilities come to mind. Visual working memory has been implicated in visual search [40, 41], raising the possibility that when visual working memory is loaded, target-distractor similarity may facilitate the chunking of objects into a searchable subset. If people were searching within a horizontal subset (e.g. [30]), then chunking would allow people to create a larger subset in visual working memory to search through. In smaller displays where a subset would include all the horizontal items, this would increase reaction times. However, in larger displays, a larger subset would increase the probability of the target being included in the subset, as well as reduce the number of subsets needed before exhausting all items in the search display, both of which would reduce search times. Another possibility is that saccades that fail to find the target, but contain non-red horizontal distractors may be priming the orientation of the target. The conditions for this to be a possibility are met, as our stimulus is relatively large, thus presumably requiring multiple saccades, and the reaction time of a single trial is long enough for the 200 ms needed for priming to take effect [37]. In other words, a saccade that failed to find the target may increase the chance of the next saccade finding the target, through priming. Both of these ideas are highly speculative though, and without solid evidence to support such interaction effects. The number of parts to either theory is also high enough to make them hard to test and falsify, putting it beyond the scope of this study.

It is also possible that the third unknown factor is a new process altogether. One process that could explain the facilitatory effect of the non-red horizontal-ish distractors comes from Bundesen’s [42] Theory of Visual Attention (TVA). TVA incorporates two main processes: filtering and pigeonholing. Filtering is a process whereby the likelihood of selecting items that belong to a category of interest is increased. It is analogous to the notion of ‘guidance’ in guided search [18, 27], or the whole process of salience based detection in signal detection models (e.g.[9, 43]). Pigeonholing is a complementary process whereby the ability to determine whether the selected item belongs to the category of interest is enhanced. In other words, it is a process of identification. It is similar to the serial stage of guided search, but does not require the separation of the processes into early and late stages. By incorporating two processes, the TVA is able to explain the facilitatory effect by postulating that people are filtering for red items, and pigeonholing the horizontal category, such that the increase in non-red horizontal-ish items means less red items overall, and pigeonholing means that any of the non-red horizontal-ish items that are erroneously selected will be rejected quickly. Since pigeonholing is a complementary process, it can be incorporated into other theories to allow them to explain the facilitatory effect.

One final point that we would like to discuss is the importance of studying complex visual search displays and the role that the genetic algorithm can have in this. As mentioned above, one of the difficulties in studying complex displays is in identifying the variables of interest among the many variables that make up its complexity. As the results of Experiment 1 show, the genetic algorithm is able to make sense of the complexity and identify variables that warrant further experimentation. While there is the limitation of being unable to interpret whether the effect is due to the variable, or an artefact of the genetic algorithm’s processes, as Experiment 2 and 3 demonstrate, this problem can be solved by supplementing the genetic algorithm with a factorial design. Another limitation that bears consideration, as illustrated in Experiment 4, is that the limited number of generations that we can realistically perform also limits the degree to which we can explore the entire problem space. This limitation is not major if one is not seeking the optimal solution to a problem, but instead seeking to understand a limited subset of the space. For example, in our case, we only sought to understand visual search in heterogeneous displays and thus continuing the evolution to homogeneous displays, which would of course produce faster search times, was irrelevant. On the other hand, a major strength of this approach is that the manipulations that the genetic algorithm makes are numerous enough that the displays appear to be changing at random, so we can be relatively sure that the effects found are not a reflection of the participants developing a certain search strategy that works due to knowledge of a manipulated variable. In other words, we can be relatively sure that we are measuring the natural allocation of the participants’ attention.

In conclusion, we have used a genetic algorithm to evolve a complex visual search stimulus and used this to study the effect of various kinds of distractors on search times. We have found visual search is hindered when a distractor shares the same colour, size and has a similar orientation to the target; or shares the same colour and orientation, but a different size to the target. We have also found visual search can be facilitated when distractors do not share the same colour as the target, but do share orientation. This facilitation effect only appears when the display is sufficiently heterogeneous. This leads us to conclude that research into heterogeneous, or otherwise complex visual search displays, may be overlooking effects that only play a small role in search for simple displays. Further, we find that the genetic algorithm provides a new methodology and a useful tool to explore possible starting points for research into complex stimuli.

## Supporting Information

S1 Dataset Dataset for the study. (XLS)

S1 Table Additional results for Experiment 1. (DOC)

S1 Text Evaluating the evolution of the genetic algorithm. (DOC)
