# Correction: Competing Distractors Facilitate Visual Search in Heterogeneous Displays

**DOI:** 10.1371/journal.pone.0173215

**Published:** 2017-02-27

**Authors:** Garry Kong, David Alais, Erik Van der Burg

The image for Fig 6 is missing the markers indicated in the figure caption. Please see the complete, correct Fig 6 here.

**Fig 6:**
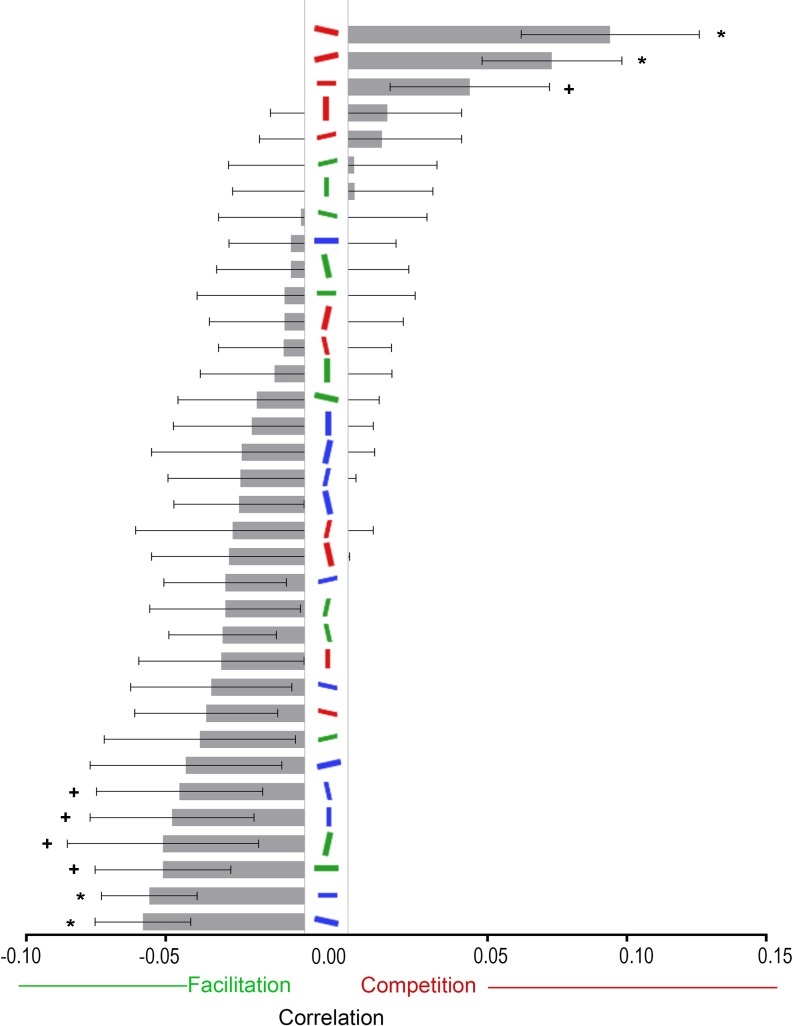
Correlations from Experiment 1 between distractor type and RTs, averaged across participants. Error bars represent ±1 within-subjects standard error of measurement [23]. * Indicates that the correlation is statistically significant at the α = .05 level after Bonferroni correction. + Indicates that the correlation is only statistically significant without Bonferroni correction.

The image for Fig 8 is incorrect. Please see the complete, correct Fig 8 here.

**Fig 8:**
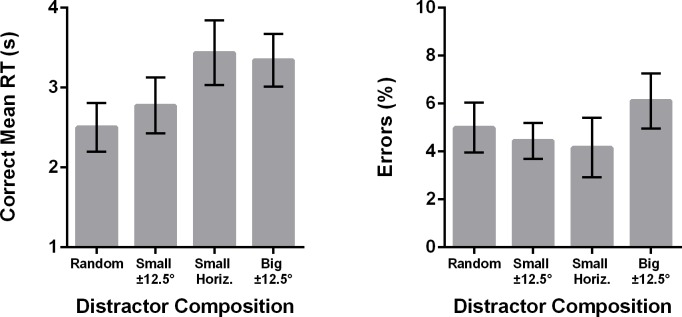
Data from Experiment 2 showing correct mean RTs as a function of the display composition. Error bars represent ±1 within-subjects standard error of measurement [23]. The x-axis indicates which of the red distractors were systematically increased in that condition. For example, small ±12.5° indicates that 20% of the display was fixed as small red 12.5° from horizontal and small red -12.5° from horizontal lines.
