# Supplementary material for: Competing Distractors Facilitate Visual Search in Heterogeneous Displays
Source: PLoS One. 2016 Aug 10;11(8):e0160914. doi: 10.1371/journal.pone.0160914 (PMC4980025; doi:10.1371/journal.pone.0160914)
Supplement: S1 Table — (DOC) [file pone.0160914.s002.doc]

S2 Supporting Information

Additional results for Experiment 1

| Size | Colour | Orientation | Mean correlation | p value |
| --- | --- | --- | --- | --- |
| Big | Red | 12.5° from H | 0.095 | 0.001 |
| Big | Red | -12.5° from H | 0.075 | 0.000 |
| Small | Red | Horizontal | 0.043 | 0.048 |
| Big | Red | Vertical | 0.014 | 0.464 |
| Small | Red | -12.5° from H | 0.013 | 0.521 |
| Big | Green | -12.5° from H | 0.002 | 0.917 |
| Small | Green | Vertical | 0.001 | 0.941 |
| Small | Green | 12.5° from H | -0.002 | 0.941 |
| Big | Blue | Horizontal | -0.005 | 0.748 |
| Big | Green | -12.5° from V | -0.006 | 0.775 |
| Big | Red | 12.5° from V | -0.007 | 0.738 |
| Small | Green | Horizontal | -0.008 | 0.702 |
| Small | Red | -12.5° from V | -0.008 | 0.627 |
| Big | Green | Vertical | -0.012 | 0.567 |
| Big | Green | 12.5° from H | -0.017 | 0.422 |
| Big | Blue | Vertical | -0.020 | 0.328 |
| Big | Blue | 12.5° from V | -0.022 | 0.236 |
| Small | Blue | 12.5° from V | -0.023 | 0.311 |
| Big | Blue | -12.5° from V | -0.024 | 0.155 |
| Small | Red | 12.5° from V | -0.026 | 0.346 |
| Big | Red | -12.5° from V | -0.028 | 0.208 |
| Small | Blue | -12.5° from H | -0.028 | 0.098 |
| Small | Green | 12.5° from V | -0.029 | 0.161 |
| Small | Green | -12.5° from V | -0.030 | 0.043 |
| Small | Red | Vertical | -0.031 | 0.169 |
| Small | Blue | 12.5° from H | -0.033 | 0.134 |
| Small | Red | 12.5° from H | -0.035 | 0.070 |
| Small | Green | -12.5° from H | -0.038 | 0.117 |
| Big | Blue | -12.5° from H | -0.043 | 0.088 |
| Small | Blue | -12.5° from V | -0.046 | 0.036 |
| Small | Blue | Vertical | -0.047 | 0.037 |
| Big | Green | 12.5° from V | -0.051 | 0.046 |
| Big | Green | Horizontal | -0.052 | 0.011 |
| Small | Blue | Horizontal | -0.056 | 0.000 |
| Big | Blue | 12.5° from H | -0.057 | 0.000 |

# Table S2.1. Exact correlation and p-value statistics for Experiment 1.
