# Supplementary material for: Competing Distractors Facilitate Visual Search in Heterogeneous Displays
Source: PLoS One. 2016 Aug 10;11(8):e0160914. doi: 10.1371/journal.pone.0160914 (PMC4980025; doi:10.1371/journal.pone.0160914)
Supplement: S1 Text — (DOC) [file pone.0160914.s003.doc]

S1 Supporting Information

Evaluating the evolution of the genetic algorithm

In this section, we will describe several ways to evaluate the genetic algorithm’s evolution. These methods will then be evaluated using a Monte Carlo Simulation. This simulation was a recreation of Experiment 1, where instead of having participants perform the visual search task, a random number was drawn from a normal distribution with a mean of 5 and a standard deviation of 1 to approximate the reaction times that the participants had. All other parameters were left as is, and the simulation was performed 5000 times. Since the ‘reaction times’ were randomly generation, the genetic algorithms should fail to evolve. Any analysis method that finds a successful evolution in more than 5% of the simulations will indicate that the method is not suitable for analysing genetic algorithms, as that would exceed our desired type I error rate of 0.05.

The simplest way in which we can analyse the genetic algorithm’s evolution is to subtract the number of times each element appeared in the first generation from the number of times it appeared last generation. A significant repeated-measures ANOVA on this generational difference would be either interpreted as a successful evolution of the genetic algorithm, or a type I error. In the 5000 iterations, 238 iterations (4.76%) produced a significant repeated-measures ANOVA. This number is in line with the desired type I error rate of 0.05 (less than 5% of the iterations produced a significant effect from bogus data) allowing us to conclude that this method is satisfactory for evaluating the evolution of a genetic algorithm.

However, this method has a conceptual problem in that it makes the assumption that the successful evolution was meaningful, i.e., that a reduction in the fit values (here reaction times) caused the evolution and vice versa. This assumption need not be made though, as this can be tested directly by correlating each element of the genetic algorithm with the relevant fit value, e.g., in our experiment we correlated the number of each element in the display with the median reaction time for that display. A significant t-test on all the correlations would, after Bonferroni correction, indicate that there was a meaningful evolution of the genetic algorithm, or a type I error. In the 5000 iterations of the Monte Carlo simulation, a significant correlation between one distractor and reaction time was found in 222 iterations, and significant correlations between two distractors and reaction time found for 2 iterations, for a total of 224 (4.48%). This too is in line with the desired type 1 error rate of 0.05.

Conceptually, doing both of the analyses described above is the soundest method. A significant change in the composition of the displays in the last generation when compared to the first, in addition to these changes being correlated with reaction time would indicate that the evolution was both successful and meaningful. In the Monte Carlo simulation, only in 14 iterations (0.28%) was a type I error observed using both analyses at the same time. Practically though, an analysis of only the correlations between the number of times a distractor appears in each generation and reaction time already sufficiently controls the type I error rate and furthermore, it would be difficult to argue that any significant correlations were not an indication of a successful evolution.

The last method to analyse the genetic algorithm’s evolution is to look for an improvement in fit value across generations. This is the most intuitive method, as one would think that the whole purpose of the genetic algorithm is to improve the fit values. While this may be true for computer simulations, this method becomes problematic when humans are involved. This is because the fit value is now not only influenced by the stimuli they are evaluating, but also by human factors such as practice and fatigue. A practice effect would cause an improvement in fit value in absence of any evolution at all.

Importantly however, such across-generation effects do not affect the success of the genetic algorithm, as the genetic algorithm compare the fit values within a generation, not across. We can model this in our Monte Carlo simulation by shifting every generation the normal distribution which we used to draw the random fit values, e.g., by subtracting 0.5 from the mean of 5 that it was set at. By doing this, we would find that the fit values decreased by 0.5 each generation, emulating a practise effect, but evolution be unaffected. The genetic algorithm selects the top 4 displays within each generation, with no regard for the results of previous generations. Thus the same 4 displays would have been selected, regardless of the mean of the distribution being 5 or 3.

An improvement in the fit value is a good sign of a successful evolution only in that it does not indicate a deterioration in fit values, which is a likely sign of unsuccessful evolution. One should not draw conclusions from an improvement in fit values and instead use one of the other methods described above. Best practise would be check for a both a significant change in the last generation when compared to the first generation, and for a significant correlation between these changes and reaction time. This is what we have done in the current study.
